# Future Directions and Molecular Basis of Ventilator Associated Pneumonia

**DOI:** 10.1155/2017/2614602

**Published:** 2017-10-15

**Authors:** Kubra Aykac, Yasemin Ozsurekci, Sevgen Tanir Basaranoglu

**Affiliations:** Department of Pediatric Infectious Diseases, Hacettepe University Faculty of Medicine, Ankara, Turkey

## Abstract

Mechanical ventilation is a lifesaving treatment and has complications such as ventilator associated pneumonia (VAP) that lead to high morbidity and mortality. Moreover VAP is the second most common hospital-acquired infection in pediatric intensive care units. Although it is still not well understood, understanding molecular pathogenesis is essential for preventing and treating pneumonia. A lot of microbes are detected as a causative agent of VAP. The most common isolated VAP pathogens in pediatric patients are* Staphylococcus aureus, Pseudomonas aeruginosa, *and other gram negative bacteria. All of the bacteria have different pathogenesis due to their different virulence factors and host reactions. This review article focused on mechanisms of VAP with molecular pathogenesis of the causative bacteria one by one from the literature. We hope that we know more about molecular pathogenesis of VAP and we can investigate and focus on the management of the disease in near future.

## 1. Introduction

### 1.1. Ventilator Associated Pneumonia

Mechanical ventilation is an essential, lifesaving therapy for patients with critical illness and respiratory failure [1]. These patients are at high risk for complications such as ventilator associated pneumonia (VAP) with a prevalence ranging from 6.6% to 32% [2, 3]. Additionally VAP is a significant problem among pediatric intensive care units due to the fact that it is the second most common hospital-acquired infection after bloodstream infections [4]. Moreover it causes an increase in morbidity, mortality, and length of stay in the hospital, particularly in intensive care unit as well as care costs [3, 5].

### 1.2. Methods

We evaluated both reviews and original articles about molecular basis of ventilator associated pneumonia, new diagnostic biomarkers, and its future directions from database of PUBMED (1986 to 2016). The keywords “molecular” or “ventilator associated pneumonia” or “bacterial pneumonia” or “biomarkers” were used. Last definitions, especially, new biomarkers, and mostly seen bacterial agents were investigated and revealed about ventilator associated pneumonia.

### 1.3. Definition and Diagnosis of VAP

There is no gold standard, valid definition, and diagnosis for VAP and even the most widely used VAP criteria and definitions are neither sensitive nor specific [1]. In 2016, Centers for Disease Control and Prevention (CDC) reported a module about the definition of VAP as follows: a pneumonia where the patient is on mechanical ventilation for >2 calendar days on the date of event, with day of ventilator placement being day 1, and the ventilator was in place on the date of event or the day before [6].

Clinical suspicion of VAP in a patient is the initial part of diagnosis.

In addition, according to CDC, the standard diagnostic criteria include [6] the following:

For any pediatric patient, at least one of the following:

- Fever > 38°C or hypothermia of <36.5°C,
- Leukopenia ≤ 4000 WBC/mm^3^ or leukocytosis ≥ 15,000 WBC/mm^3^,

And at least two of the following:

- New onset of purulent sputum or change in character of sputum or increased respiratory secretions or increased suctioning requirements,
- New onset or worsening cough or dyspnea or tachypnea,
- Rales or bronchial breath sounds,
- Worsening gas exchange (e. g., O_2_ desaturations [e.g., PaO_2_/FiO^2^ ≤ 240], increased oxygen requirements, or increased ventilator demand),

At least three (only for child > 1 year old or ≤12 years old) of the following:

- Fever of >38°C or hypothermia of <36.5°C,
- Leukopenia ≤ 4000 WBC/mm^3^ or leukocytosis ≥ 15,000 WBC/mm^3^,
- New onset of purulent sputum or change in character of sputum or increased respiratory secretions or increased suctioning requirements,
- New onset or worsening cough or dyspnea, apnea, or tachypnea,
- Rales or bronchial breath sounds,
- Worsening gas exchange (e. g., O_2_ desaturations [e.g., pulse oximetry < 94%], increased oxygen requirements, or increased ventilator demand),

At least three (only for infants ≤ 1 year old) of the following:

- Temperature instability,
- Leukopenia ≤ 4000 WBC/mm^3^ or leukocytosis ≥ 15,000 WBC/mm^3^ and left shift (≥10% band forms),
- New onset of purulent sputum or change in character of sputum or increased respiratory secretions or increased suctioning requirements,
- Apnea, tachypnea, and nasal flaring with retraction of chest wall of nasal flaring with grunting,
- Wheezing, rales, or rhonchi,
- Cough,
- Bradycardia (<100 beats/min) or tachycardia (>170 beats/min) [6].

The presence of at least one of the following on one or more (in patients with underlying diseases two or more) serial chest radiographs, new or progressive radiographic infiltrates, consolidation, cavitation, and pneumatoceles in an infant ≤ 1 year old will also be needed for the diagnosis [6].

Besides physical examination, culture is important because it can establish the causative organism and guide the treatment. Culture specimens can be obtained by tracheal aspirate or bronchoalveolar lavage [4]. However more studies should be performed to achieve the most reasonable culture results [7].

Current studies tend towards to biological markers in the diagnostic algorithm of VAP. It was reported that soluble triggering receptor expressed on myeloid cells-1 and surfactant protein-D level of bronchoalveolar lavage fluid and serum procalcitonin levels might be useful predictors for VAP [8–10]. There are a plenty of studies about the risk factors of VAP. Reintubation, presence of tracheostomy, enteral feeding, prolonged PICU, or hospital stay regardless of illness severity, genetic syndrome, transport out of the PICU, positive blood culture, prior antibiotic usage, bronchoscopy, immunodeficiency, immunosuppressant drugs, neuromuscular blockade, gastric aspiration, mechanic ventilation longer than 3 days, the use of acid-suppressive therapy, neuromuscular diseases, histamine-2 receptor blockers, vasoactive drugs, and presence of a nasoenteral tube were reported to be related to increased risk for VAP [2, 11–16].

## 2. Molecular Pathogenesis of Pneumonia

Understanding molecular pathogenesis of pneumonia is essential to prevent and treat pneumonia; however it is still not well understood. Nowadays the studies showed that uncontrollable epithelial cell death is fundamental to the pathogenesis of pneumonia especially in early stages of inflammation. In an animal study, Zou et al. discovered expression of a protein named the mortality factor 4 like 1 (Morf 4l1) increases in humans with pneumonia and it is related to host cell death during pulmonary inflammation [17]. Cytokine response, neutrophil activity, and responsiveness to cytokines and neutrophil lifespan are important in lung infection pathogenesis. The degree of neutrophil activation, generation of reactive oxygen species, and the release of granule proteins are significant in microbial pathogen clearance [18]. Cytotoxins such as *α*-hemolysin (HIa) are also very important in the pathogenesis. HIa is highly potent in lysing bronchial and alveolar epithelial cells, macrophages, and lymphocytes and it is official in proinflammatory processes [19].

## 3. Pathogens

A lot of microbes were detected as a causative agent of VAP. The most common isolated VAP pathogens in pediatric patients are* Staphylococcus aureus, Pseudomonas aeruginosa, *and other gram negative bacteria [3, 7]. Nonetheless, most of the tracheal isolates from patients with VAP were polymicrobial [7]. All of these pathogens cause VAP by using patients' weakened lung defence systems, resulting from pulmonary and systemic illness and medical therapy, worsening the normal host microbial flora by illness, antibiotics, and mechanic ventilation devices [20].

Initially we should use an antimicrobial therapy which covers the bacteria confirmed by the culture for the treatment rather than broad spectrum antibiotics because of avoiding of antimicrobial resistance of VAP [4]. Antimicrobial-coated endotracheal tubes (EET) are still an investigating target due to the fact that bacteria create biofilm on ETTs and then enter the lungs and cause pneumonia. There are several studies on EETs [21–23]. In 2015 Tokmaji et al. published a review about silver-coated EETs for prevention of VAP and stated limited evidence in reduction of the VAP risk [21]. It is showed by using scanning electron microscopy that gardine- and gendine-coated ETT inhibited the formation of biofilms due to bacteria and were found to be more effective in preventing biofilm growth than the silver ETTs. Nevertheless, several animal and clinical studies are needed to validate the efficacy and safety [23].

### 3.1. *Pseudomonas aeruginosa*

Secondary nosocomial pneumoniae is one of the frequent causes of deaths in hospital setting in spite of the developing medicine [24] and one of the major causative bacterial pathogens is* Pseudomonas aeruginosa *with an extremely high mortality rate, particularly in patients with impaired immunity as an opportunistic pathogen [25, 26]. Therefore, there is an increasing interest in the use of immunoadjuvant therapy to recruit host immunity in patients with such kinds of infections. IL-17 stimulates proliferation of cells in the lymphoid lineage and has crucial roles for survival, development, and homeostasis of lymphocytes [27–29]. IL-17, its T receptor pathway, and TNF-*α* also have important roles in pulmonary clearance of gram negative bacteria and thereby improving survival [29]. Therefore, recombinant human IL-7 (rhIL-7) is one of the most promising of these new immunoadjuvants and has been effective in decreasing mortality in animals with immunosuppressive state [30].

Shindo et al. [29] demonstrated that rhIL-7 improves survival in secondary* P. aeruginosa *pneumonia. Moreover, they showed the increase in the production of lymphocytes that secrete IFN-*δ*, IL-17, and TNF-*α*, cytokines that are important in the host defence against both sepsis and pneumoniae caused by* P. aeruginosa.* The increase in those cytokines is important because Pastille et al. [31] stated in an animal model that a disturbed interaction of accessory cells and NK cells may be the cause of the damaged release of IFN-*δ* in mice treated with* Pseudomonas. *Early production of IL-17 seems play a crucial role in infected mice with* P. aeruginosa *despite the fact that the exact role of IL-17 is unclear [32].

Pneumoniae caused by* P. aeruginosa* is a complex process. Some membrane surface elements including flagellum, fimbriae, and polysaccharides are used via a number of aggregated pili to adhere to respiratory epithelia [33, 34]. Immune response of the host is stimulated by means of some virulence factors such as type III secretory protein, quorum-sensing system, and lipopolysaccharides (LPS). Consequently, secreted cytokines, chemotactic factors, and other inflammatory mediators by host cells cause severe lung injury and mortality through activating macrophages, neutrophilic granulocytes, and T cells [35, 36]. Phosphoinositide 3-kinase (PI3K)/AKT and Ras/Mitogen activated protein kinase (MAPK) have significant roles in the membrane receptors signalling, which are involved in both the inflammatory and immune responses [37, 38]. LPS activates protein kinase-C (PKC) and initiates proinflammatory signals such as focal adhesion kinase (FAK), a protein tyrosine kinase, and Ras, a GTPase. PI3K and MAPK activated by FAK and Ras activate ERK and p38 to regulate the expression of cytokines in the immune and inflammatory responses [39–42]. PI3K and MAPKs may be activated by cytokines besides other growth factors to arrange the proliferation and differentiation of cells and the expression of proinflammatory factors [43]. Hou et al. designed a study using TNF-*α* induced cultured cells to ascertain the anti-inflammatory effect of Qingfei Xiaoyan Wan, a traditional Chinese medicine formula, in animals with* P. aeruginosa *induced acute lung inflammation. They showed that several ingredients of this drug, arctigenin being the principal one, have potential suppression on the primary intracellular immune response and affect the pathways of PI3K/AKT and Ras/MAPK [44].

### 3.2. * Staphylococcus aureus*

*S. aureus* is one of the most common pathogens associated with VAP in the PICU [45]. In a prospective study, 26 children with VAP were investigated and* S. aureus* was detected in 28.4% of patients with the highest rate [7]. Generally most strains are methicillin-sensitive* S. aureus, *but the prevalence of methicillin-resistant strains is increasing, even in community isolates [20]. There are several factors of being respiratory diseases, such as substantial metabolic capabilities, genetic flexibility, ability to adapt to environmental pressures, and exploitation of the immune responses that are evoked [46].

Virulence factor expressions are controlled by a number of regulatory systems in the genome of* S. aureus* such as accessory gene regulatory (Agr) system. This system regulates the expression of surface proteins (protein A) and secreted toxins (toxic shock syndrome toxin, hemolysin, and staphylokinase) [47]. Agr and its regulation are necessary for invasive pulmonary infection. The expression of Agr is required for intracellular life to escape from endosomes.* S. aureus *can live for long time in neutrophils and in some cases divide within dying cells; thus, this can contribute to a systemic dissemination of the organisms. Due to these features, development of vaccines seems unlikely to be successful. *δ* and *β* toxin act to facilitate staphylococcal escape from endosomes in airway epithelial cells [46]. Additionally the lung with increased airway permeability, inhibition of ciliary beat frequency, and neutrophilic response are associated with *β*-toxin, that is, a sphingomyelinase which targets the membranes of host cells [48–50]. Similarly *α*-toxin is essential for the pathogenesis of* S. aureus *pneumonia. Qiu et al. studied on isoalantolactone a natural compound from* Inula helenium* (IAL) against* S. aureus* in vitro and reported that IAL can markedly inhibit the expression of *α*-toxin at very low concentrations [51]. In other studies about *α*-toxin, it is stated that capsaicin and silibinin have prevented *α*-toxin-mediated alveolar cell injury through animal studies about pneumonia caused by* S. aureus* [52, 53].

Panton-valentine leukocidin (PVL) is a toxin encoded by lukF-PV and lukS-PV genes carried on a bacteriophage. PVL causes the apoptosis of neutrophils via caspases 3 and 9 and inflammation and contributes necrotizing pneumonia [46]. New studies about anti-PVL monoclonal antibodies might be useful for early diagnosis and treatment [54]. For diagnosis of* S. aureus*, Linge et al. suggested that one of the antimicrobial protein midkine was detected in sputum from patients suffering from VAP caused by* S. aureus* [55]. Stulik et al. published their data that airway colonization with MSSA strains with high HIa is a predictor of progression to VAP. Moreover HIa is currently being evaluated for using as active and passive immunization in human trials [19]. Recently, Achouiti et al. investigated host response in mice and found that* S. aureus* pneumonia was associated with a strong rise in myeloid-related protein 8/14 in bronchoalveolar lavage fluid and lung tissue [56].

These studies support reasonable approach to antivirulence factors as a new antibacterial target for treatment.

### 3.3. *Klebsiella pneumoniae*

*Klebsiella pneumoniae *is the causative agent of a wide range of infections including pneumonia, bacteremia, and sepsis and is the third most common cause of hospital-acquired infections [57]. This bacteria is rapidly acquiring resistance to all known antibiotics, including carbapenems [58]. Multidrug resistant* K. Pneumoniae *exhibits about 50% of mortality rate in patients with bloodstream infections [59, 60].* K. pneumoniae *has acquired carbapenemases, which are enzymes capable of breaking down most *β*-lactams, including* K. Pneumoniae *(KPCs), carbapenemases of the oxacillinase-48 (OXA-48), and New Delhi Metallo-*β*-lactamase (NDM) carbapenemases have important roles in the rapid dissemination of the disease [61]. Various class carbapenemases have been identified worldwide in* K. pneumoniae *as well as hospital-acquired multiresistant* K. Pneumoniae* [62, 63].

*K. pneumoniae *siderophores are big contributing factors in the inflammation and bacterial dissemination of the patients with lung infection caused by* K. pneumoniae *according to data of Holden et al. [64]. Additionally, they also showed that master transcription factor hypoxia inducible factor-1 *α* (HIF-1 *α*) is needed for bacterial dissemination in lung epithelial cells. Siderophores, high-affinity iron chelating molecules, have important role for bacterial growth and replication in many gram negative bacteria including* K. pneumonia* [65–67]. Although siderophore-associated iron regulation during bacterial infections is relatively unclear, iron chelation via siderophores induces cytokine secretion of interleukin-8 (IL-8), IL-6, and chemokine (C-C motif) ligand 20 from lung epithelial cells [64, 67]. Siderophores stabilize HIF-1 *α* in vitro [68, 69]. HIF-1 *α* regulates the function of many genes that have some critical roles in glycolysis, inflammation, and angiogenesis [70]. HIF-1 *α* activation has been associated with innate immunity against infections [71].

The invasive nature of* Klebsiella* may be attributed to* K. pneumonia *strain, expressing the K1 or K2 capsular antigen [72]. K1/K2 serotype* K. pneumonia *strains are highly pathogenic because of the presence of mucoviscosity-associated gene a (mag A), regulator mucoid phenotype A gene (rmpA), and capsular antigens K1, K2. Those factors induce resistance to phagocytosis through neutrophils and macrophages that are involved in the early innate immune response to intrapulmonary* K. pneumonia *infection [72, 73].

CD36, a scavenger receptor that has a role in the innate immune response to* K. pneumonia *and recognizes pathogen and modified self-ligands, is a host determinant of* K. pneumonia *pathogenicity and mainly expressed via some cell types including macrophages, endothelial cells, and epithelial cells [74]. Olonisakin et al. [75] showed the critical role of CD36 for optimal control of* K. pneumonia *in the lungs and extrapulmonary bacterial dissemination by mediating recognition of LPS, enhancing alveolar macrophage activity, and managing the optimal cytokine production in the lungs in an acute bacterial pneumonia model. Additionally, CD36 also regulates TLR4/TLR6 complex formation to potentiate NF-kB activity [76].

Chemokines, secreted locally or in paracrine and autocrine fashions by leucocytes and tissue cells, aside from chemokine receptors are crucial therapeutic targets for the diseases [77]. CXC chemokines (alpha chemokines) are subdivided according to presence of the Glu-LeuArg (ELR) tripeptide motif. The ELR-CXC family of chemokines regulates neutrophil recruitment and CXCL8 (interleukin (IL8)); the prototypical ELR-CXC chemokine is commonly detected in infections caused by* Klebsiella* [78]. Lungs endothelial cells primarily secrete ELR-CXC chemokines and other inflammatory mediators following bacterial activation. A human CXCL8 analogue (G31P), antagonizes both CXCL1 and CXCL2, seems a promising agent in some models such as aspiration pneumoniae and ischemia-reperfusion injury [79, 80].

### 3.4. * Acinetobacter baumannii*

*A. baumannii* is increasingly becoming one of the most common pathogens causing VAP [81]. There are very few studies about genetic molecular basis of* A. Baumannii* infections. Elhosseiny et al. investigated universal stress protein A (UspA) in* A. baumannii* pneumonia in animals. They highlighted the role of UspA as an important contributor to the* A. baumannii* virulence and it could be a new therapeutic target [82]. In another animal study phospholipase D seems to be an* A. baumannii* virulence factor [83]. In 2015, Méndez et al. suggested that ex vivo proteome of* A. baumannii* is an important step for diagnostic biomarkers, novel drug targets, and potential vaccine candidates against* A. baumannii* pneumonia [84].

Recently, a connection between host-mediated metal starvation and metabolic stress in* A. baumannii *pneumonia was reported in a new study published about antimicrobial activity of calprotectin [85]. Moreover, a tumor suppressor protein recently described as immunoregulatory protein Fus1 has a role in the immune response to* A. baumannii* and Hood et al. stated that this could be a new avenue for immune modulating therapeutic targets [86]. In 2016, it was reported that immunization with an outer membrane nuclease (NucAb) decreased bacterial load, cytokines, and inflammation in mice lungs and, thus, it could be a vaccine candidate in* A. baumannii* infection [22]. Nowadays, treating multidrug resistance of* A. baumannii* with currently available drugs is difficult; that is, we should start investigating vaccines besides new drugs.

### 3.5. * Escherichia coli*

*E. coli* is the main Enterobacteriaceae caused VAP [87]. Due to increasing multidrug resistance among* E. coli*, scientists investigate genotypic and phenotypic characteristics of* E. coli* and aim to discover immunotherapy and vaccine against* E. coli* [88]. Dufour et al. studied the effect of bacteriophage treatment on mice and reported that phage therapy could be a promising therapeutic strategy for VAP [89].

The clearance of microbes from respiratory tract requires systemic and localized inflammatory response controlled by host-derived cytokines [90]. Alveolar epithelial STAT3 activated by IL-6 family members functions to promote neutrophil recruitment and limits infection and injury during* E.coli* pneumonia [91]. Therefore, Cui et al. tested the proinflammatory effects of TGF-*β*1 in* E. coli *pneumonia and stated that TGF-*β*1 was associated with improved microbial clearance in rat models of pneumonia while overall survival was not significantly improved [92]. In addition, it was shown that deficiency of tissue-expressed CD47 (integrin associated protein) protects the lung parenchyma whereas deficiency of CD44 (cell-surface receptor for hyaluronic acid) leads to lung injury in* E. coli* pneumonia in mice [93, 94]. In 2010, an endogenous mediator called resolvin E1 was presented for the first candidate as a novel therapeutic for acute lung injury and pneumonia due to* E. coli* with an animal study [95]. There are not many studies about molecular activities of* Enterobacter* spp. Kostiushko and Markelova investigated the cytokine profiles at the experimental* Enterobacter* pneumonia and reported that local levels of cytokines are different from pneumonia caused by* E. coli* and* Enterobacter spp*. [96].

## 4. Conclusion

There are continuing rapid advances in our understanding of the basic mechanisms of VAP. Understanding of these mechanisms may guide the discovery of the possible therapeutic targets for improving host defence, preventing lung injury and infection. Because of increasing antimicrobial resistance and scarcity of new antibiotic discovery there should be more studies about mechanisms of VAP for finding new approaches for prevention and treatment.
